# A highly effective mindfulness intervention for burnout prevention and resiliency building in nurses

**DOI:** 10.3934/publichealth.2025007

**Published:** 2025-01-14

**Authors:** Maryanna Klatt, Jacqueline Caputo, Julia Tripodo, Nimisha Panabakam, Slate Bretz, Yulia Mulugeta, Beth Steinberg

**Affiliations:** Center for Integrative Health, Department of Family and Community Medicine, The Ohio State University College of Medicine, Columbus, Ohio, USA

**Keywords:** burnout, resilience, mindfulness-based interventions, organizational interventions, healthcare worker burnout

## Abstract

**Objectives:**

Healthcare workers, most notably nursing professionals, face high levels of recurrent stress that lead to symptoms of burnout and other negative mental health outcomes. This indicates the need for greater individual and organizational health system support, including implementation of effective, evidence-based interventions for burnout reduction in this population. Organizationally supported mindfulness-based interventions can be used to build individual resilience, buffering the detrimental effects of occupational stress and enhance professional well-being. Our aim was to evaluate the effect of an evidence-based mindfulness intervention on perceived stress, burnout, resilience, and work engagement in nursing professionals.

**Methods:**

A non-randomized single arm, pre/post design was used for this study. Healthcare workers (*n* = 631), including Registered Nurses and Advanced Practice Nurses (*n* = 128), physicians (*n* = 105), social workers (*n* = 21), pharmacists (*n* = 8), chaplains (*n* = 30), physician and occupational therapists (*n* = 39), patient care assistants (*n* = 13), other clinical staff (*n* = 26), non-clinical staff (*n* = 229), and 32 others participated in Mindfulness in Motion, an 8-week evidence-based worksite mindfulness intervention. Validated self-report measures assessing burnout (Maslach Burnout Inventory), perceived stress (Perceived Stress Scale), resilience (Connor Davidson Resiliency Scale), and work engagement (Utrecht Work Engagement Score) were given pre and post program. Total burnout was determined by scores on the subscales of emotional exhaustion, depersonalization, and personal accomplishment of the Maslach Burnout Inventory: Emotional exhaustion >27 or depersonalization >13 or personal accomplishment <31.

**Results:**

Significant reductions in burnout (*p* < 0.00001), perceived stress (*p* < 0.00001), with significant increases in resilience (*p* < 0.00001), and work engagement (*p* < 0.00001) were observed among Registered and Advanced Practice Nurses comparing pre-post measures. Notably, the number of nursing professionals who no longer qualified as burned out was 10% higher than the other participants.

**Conclusion:**

Our results suggest that nursing professionals could greatly benefit from organizationally supported mindfulness-based interventions such as Mindfulness in Motion and may benefit them most compared to other health system employees.

## 1. Introduction

### 1.1. Burnout in nursing personnel

Healthcare workers (HCWs), particularly nurses given their high demands regarding patient care, are highly subject to burnout and its impact on mental health and well-being [1]. Nurses worldwide often experience high levels of occupational fatigue and psychological stress in various forms [2]–[5]. These symptoms can lead to burnout, which can be the precursor for compassion fatigue, sleep disruptions, and other mental health disorders. Consequences of burnout may also include occupational injury, increased risk of substance use, decreased mental health, reduced professional development, and challenges in managing workplace stress, while maintaining compassion for oneself and others [6].

While the symptoms of burnout are experienced individually, burnout may stem from systematic issues within a healthcare organization. An individual may experience burnout when there is a discrepancy between job demands and available supportive resources. A recent study conducted with 121 hospital-based nurses was undertaken to determine if nurse burnout was influenced by individual nurse characteristics and/or work environment factors. This sample of educated, tenured, and predominantly female nurses completed the Maslach Burnout Inventory (MBI) and the Copenhagen Psychosocial Questionnaire III (COPSO III) short version. Structural Equation Model (SEM) analysis found that individual characteristics of the nurses including age, gender, marital status, education, and tenure, were not associated with the dimensions of burnout, specifically emotional fatigue, depersonalization, or impairment of achievement. However, the psychosocial aspects of the work environment including job demands, work interactions, perceived lack of control, and perceptions of leadership support, were significantly associated with the dimensions of burnout for these nurses [7]. Emotional exhaustion, which is a key factor influencing burnout, is directly impacted by job demands, and in turn, is related to safety performance [7]. Patient safety is paramount in patient care and effective healthcare delivery; thus, organizations have a vested interest in ensuring that nursing professionals remain psychologically and physically healthy. Resilience programming delivered via the organization may be an avenue to mitigate the job stresses for nursing professionals. Because psychosocial safety climate, job demands, and job satisfaction are directly related to safety performance, it is critical that nursing professionals are given the tools to care for themselves on the job [8].

The COVID-19 pandemic highlighted the importance of providing resiliency building tools to those in the nursing profession. Healthcare workers (HCWs), including nurses, who worked directly with patients suffering from COVID-19 showed higher rates of depression, anxiety, PTSD symptoms, and burnout than their counterparts who did not directly treat COVID-19 patients [9]. Post-COVID, nurses continue to suffer from burnout, exacerbated by staffing shortages, demanding workloads, and limited organizational resources [10]. With the goal of addressing burnout in a group of nurses working in cardiovascular acute care units, a recent study that included a mindfulness-based smartphone application was implemented and evaluated as an intervention to address burnout in 31 inpatient nurses who worked in an academic medical center. Understanding that the COVID-19 pandemic had worsened work-related stress for these inpatient nurses, the nurses were asked to spend five minutes per day using the smartphone app at work or home for 30 days. Findings included significant reductions in burnout as measured by the Copenhagen Burnout Inventory and significant reductions in perceived stress as measured by the Perceived Stress Scale. The authors concluded that the implementation of brief, on-demand mindfulness practices could be considered as a form of self-care for busy inpatient nurses [10]. In this study, we highlight that pragmatic, data-driven burnout prevention and resiliency building interventions are essential and can be effective.

### 1.2. A model to address burnout

**Figure 1.**
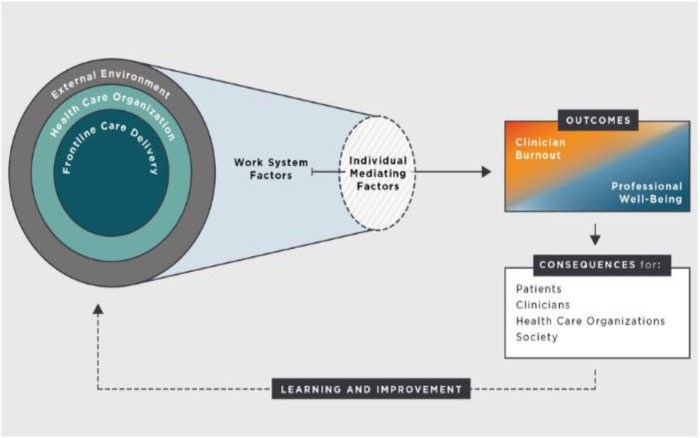
Systems model of clinician burnout and professional well-being [11].

The *Systems Model of Clinician Burnout and Professional Well-being* is a conceptual framework developed by the Committee on Systems Approaches to Improve Patient Care by Supporting Clinical Well-being at the National Academies of Sciences, Engineering, and Medicine. This framework suggests that there are factors including the external environment, the health care organization, and care delivery systems that can lead to clinician burnout or promote clinician well-being [11]. The model also proposes that there are individual mediating factors that determine an outcome of burnout or of well-being [11]. Either outcome results in downstream effects for the individual clinician, work colleagues, the organization, and society (Figure 1) [11].

Per Shanafelt [12], health care leaders must align to an organizational strategy that focuses on “cultivating well-being and preventing occupational distress” in the healthcare workforce. This includes a coordinated effort to minimize or eliminate system-level stressors and facilitate the development and implementation of evidence-based individual interventions that may help support clinician health and well-being. As a pragmatic approach to lessen the significant well-being concerns within the nursing profession, healthcare systems can target the individual mediating factors by teaching resilience building and stress management techniques, concurrently with system change.

In 2020, the Coping with COVID-19 survey, distributed to 208 U.S. healthcare organizations, was completed by 37,685 physicians, advanced practice nurses, registered nurses, and other clinical staff. Healthcare workers offered perceptions of burnout, intent to leave, and feeling valued by the healthcare organization. Qualitative comments about the work environment, work stressors, leadership, and compensation were also collected [13]. The results showed that clinical staff who felt highly valued by their organization had 8.3 times lower odds of burnout and 10.2 lower odds of intent to leave the organization [13]. This research also detailed that only 45% of participants felt valued, highlighting the urgent need for effective system and individual level interventions that can mitigate this crisis.

### 1.3. Mindfulness as an approach to address burnout

Mindfulness-based interventions (MBIs) can help individuals feel valued and have gained support and acceptance to reduce stress and improve work life quality for nurses. These relatively low-cost interventions can be adapted and easily integrated into a variety of health care settings, offering psychological and well-being support [14],[15]. When endorsed and supported by the health care organization, the availability and accessibility of worksite MBIs for HCWs, as an integral intervention to support health and well-being, reflects a commitment to an organizational culture of well-being [12].

Mindfulness-based interventions are modified versions of the Mindfulness-Based Stress Reduction (MBSR) program. Developed over 40 years ago by Jon Kabat-Zinn from the University of Massachusetts Medical School, MBSR was offered to assist and support the ongoing care of patients with chronic pain that was resistant to traditional medical interventions [14]. His ongoing work of treating these patients with a standardized program of therapy has been chronicled in multiple books and peer reviewed papers [14]–[18]. Simply defined, mindfulness is a non-judgmental awareness and acceptance of the present moment or circumstances. Mindfulness-based interventions encourage introspection and appreciation, allowing one to non-judgmentally reframe perceptions and understanding of events [14]. Mindfulness-based Stress Reduction is an eight-week program that consists of 2.5-hour weekly sessions and a recommendation for 45 minutes of daily practice [14],[19]. While time intensive, years of evidence support significant decreases in physical and psychological symptoms of stress in patients. Adapting MBSR, typically by shortening the length of the weekly sessions and recommendations for daily practice, has been found to offer similar benefits for different populations, such as HCWs [14],[18]. These adaptations are essential when designing a program that can fit into the busy clinical workflows of HCWs, especially nursing professionals.

Mindfulness in Motion (MIM) is one such evidenced-based, organizationally sponsored MBI that has consistently delivered significant reductions in HCW burnout and perceived stress, with significant increases in resilience and work engagement [20]. Mindfulness in Motion is an eight-week evidence-based program specifically designed as a workplace intervention to help HCWs learn practical burnout/stress reduction and resiliency building techniques. Delivered in a group format (originally in-person, now virtually), MIM includes didactic instruction, community-building group discussion, mindfulness, and gentle yoga. Each MIM session focuses on a specific theme and the protocol for HCWs has been published [21]. MIM was first implemented as a small randomized-controlled trial with 48 HCWs [22]. The program was then tested in a variety of additional studies with the hospital's faculty and staff; physician residents, and specifically surgical intensive care nurses [20],[23]–[25]. All trials showed significant results for stress and burnout reduction with improved resilience and work engagement. Biological reactions to stress, such as salivary α-amylase as markers of sympathetic activation and plasma cytokines as markers of inflammation, also decreased [26],[27]. Since 2017, over one thousand HCWs have completed MIM, sponsored by the larger health care organization, with many reporting both personal and professional benefits in the short- and long-term. Qualitative evidence has consistently shown that HCWs feel valued by the organization providing the MIM intervention. Moreover, evidence indicates that significant improvements in burnout, stress, and resilience obtained by MIM are sustained 14 months beyond program end [28].

Our goal of this study was to investigate the effect of MIM on HCWs and to compare a subset of HCWs (registered nurses and advanced practice nurses) to other HCWs. We are the first to examine nursing personnel separately from the larger group of HCWs who have participated in MIM, which is crucial to understanding how healthcare leadership can maintain employee satisfaction among the largest group of health professionals within a health system.

## 2. Methods

The study design was a non-randomized single arm, pre/post study at The Ohio State University Wexner Medical Center (OSUWMC). Study approval (study number 2017B0321) was obtained by The Ohio State University Institutional Review Board prior to participant recruitment.

### 2.1. Participants

All employees at a large midwestern medical center were eligible to participate in this research study if they met the follow criteria: Aged 18 years or older, could speak, write, and understand English, and had access to a computer or mobile device that connected to the internet to have access to our website or mobile phone app. To qualify for the specific nursing analysis, all previously mentioned criteria were met, in addition to being employed as a nurse or an advanced practice nurse. Upon first log-in to the website or app, participants were asked if they would like to consent to the researchers using their aggregate data in study analysis, in addition to program improvements, or just for program improvement use. We analyzed only nursing staff data who consented for analysis and reflects nursing staff that participated in MIM from 2018–2024.

### 2.2. Intervention

Mindfulness in Motion is an eight-week evidence-based mindfulness-based intervention, specifically designed as a workplace intervention to assist HCWs learn practical coping strategies to help reduce stress and burnout and improve resilience and work engagement. Participants attended weekly one-hour synchronous sessions led by an experienced mindfulness facilitator. Each weekly session included didactic instruction, community-building group discussion, mindfulness practices, and gentle yoga. Participants were highly encouraged to engage in 5–10 minutes of individual self-practice at least five times per week (originally via a password protected website, and currently via a password protected mobile app) to further reinforce and integrate MIM practices into their work and personal lives.

### 2.3. Measures

HCWs participating in the MIM intervention were given surveys pre/post intervention. A pre-survey via a password protected website/mobile app was given one week prior to the first session to obtain general demographic information and establish a baseline on four scales: Maslach Burnout Inventory (MBI), Connor Davidson Resilience Scale (CD-RISC), Perceived Stress Scale (PSS), and Utrecht Work Engagement Scale (UWES-9) [29]–[32]. Then, at the conclusion of the 8-weeks, a post-test with the same four measures was given, along with a program evaluation that enabled qualitative comments and feedback about the MIM program (Table 2). The representative quotes show that nursing professional participants believe that the MIM intervention is an impactful organizational initiative that impacts patient care.

### 2.4. Maslach Burnout Inventory (MBI)

The MBI is a 22-item scale that measures burnout reduction in healthcare professionals and includes three subsections of burnout: emotional exhaustion (EE), personal accomplishment (PA), and depersonalization (DP), on a 0 (never) to 6 (everyday) scale. Cronbach's α was 0.90 for emotional exhaustion, 0.79 for depersonalization, and 0.71 for personal accomplishment [29]. Burnout was defined as meeting the following criteria on one or more subscales: Score ≥ 13 on depersonalization subscale, score ≥ 27 on emotional exhaustion scale, and score ≤ 31 on personal accomplishment subscale [33].

### 2.5. Perceived Stress Scale (PSS)

The PSS is a ten-item scale that evaluates the degree to which an individual perceives situations in their life to be stressful during the last month on a 5-point scale [30]. Cronbach's α was 0.90 for this scale.

### 2.6. Connor-Davidson Resilience Scale (CD-RISC)

CD-RISC is a ten-item measure of resilience which corresponds to the ability to maintain good functioning in the face of adversity [31]. Respondents rated each item from 0 (not true at all) to 4 (true nearly all the time). CD-RISC demonstrated good internal consistency (Cronbach's α value of 0.85) and validity.

### 2.7. Utrecht Work Engagement Scale (UWES-9)

UWES-9 is a nine-item questionnaire developed to measure work engagement, defined as an individual's state of fulfillment in relation to their work, characterized by dedication, absorption, and vigor [32]. Respondents rated each item from 0 (never) to 6 (always). UWES-9 demonstrated good internal consistency with Cronbach's α of 0.92 for the total score, 0.86 for vigor, 0.86 for dedication, and 0.79 for absorption subscales [32].

### 2.9. Statistical analysis

The demographics reported for nursing professions were age, length in career, gender, race, marital status, average financial stress, ethnicity, highest education level, and type of shift worked. Age is reported as a continuous variable as an average with standard deviation. All other demographics are reported as categorical data. The pre to post-test analysis was conducted using a paired samples *t*-test, with statistical significance reported as a p-value of less than 0.05. The statistical software used is STATA/SE Version 17.0.

## 3. Results

**Figure 2.**
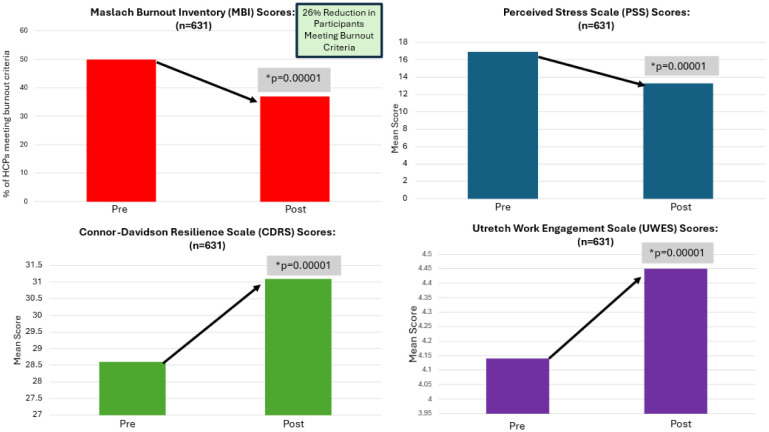
All HCWs pre to post MIM analysis for burnout, perceived stress, resilience, and work engagement.

A total of 631 HCWs completed the MIM program, completed both pre and post program surveys, and provided consent to have their aggregate data analyzed for this study. Total burnout was determined by scores on the subscales of emotional exhaustion (EE), depersonalization (DP), and personal accomplishment (PA) of the Maslach Burnout Inventory (MBI), such that a participant was considered burnt out if EE > 27 or DP > 13 or PA < 31. By the end of the eight-week intervention, there was a significant decrease in the emotional exhaustion (*p* < 0.00001) and depersonalization score (*p* < 0.00001), with a significant increase in personal accomplishment (*p* < 0.00001). All HCWs participating in the MIM program demonstrated a significant 26% reduction in burnout (*p* < 0.00001). Additionally, resilience, as measured by the CD-RISC significantly increased (*p* < 0.00001). There was also a significant increase (*p* < 0.00001) in work engagement as measured by the total UWES score, and a significant decrease in perceived stress as scored by the PSS (*p* < 0.00001). Figure 2 displays the results for the 631 HCWs.

**Figure 3.**
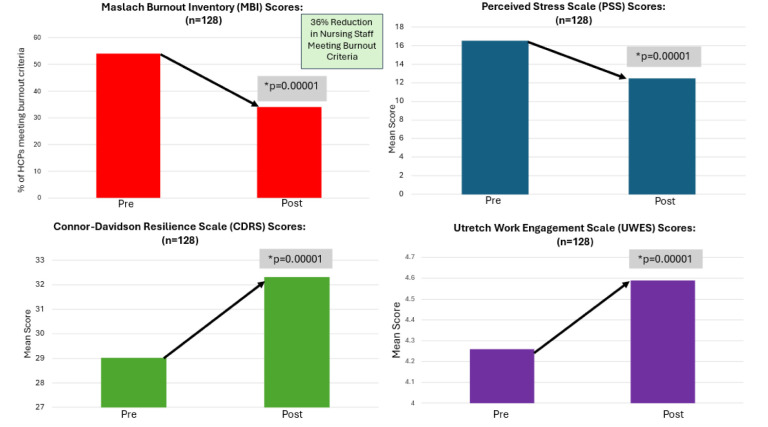
Nursing professionals pre to Post MIM analysis for burnout, perceived stress, resilience, and work engagement.

For the 631 HCWs with full data sets, 128 met the specific criteria to quality for the nursing analysis. The average age of nursing staff was 43.77 (*SD* = 11.98). The majority (42.19%) of nurses in the sample had 6–15 years experience, 21.10% had 26–34 years of experience, 14.84% had less than 5 years of experience, 12.5% of nurses had 16–25 years of experience, and the least number of nurses (9.38%) had over 35 years of experience. Nursing staff who completed the MIM program demonstrated a significant 36% reduction in burnout (*p* < 0.00001). On the subscales for the MBI, nursing staff had a significant decrease in emotional exhaustion (*p* < 0.00001) and depersonalization (*p* < 0.0003), with a significant increase in personal accomplishment (*p* < 0.00001) as compared to baseline. Resiliency significantly increased (*p* < 0.00001). There was also a significant increase (*p* < 0.00001) in the total work engagement score and a significant decrease in perceived stress scores (*p* < 0.00001). The demographic data for this group is reported in Table 1, and the results are shown in Figure 3.

**Table 1.** Demographic characteristics of the participating registered and advanced practice nurses.

**Variables**		***N* (128)**	**%**
Gender			
	Male	5	3.91
	Female	122	95.31
	Prefer not to Say	1	0.78
Race			
	American Indian or Alaska Native	2	1.56
	Asian	2	1.56
	Black or African American	6	4.69
	White or Caucasian	115	89.84
	More than one race	3	2.34
Marital Status			
	Married	88	68.75
	Divorced	16	12.50
	Widowed	2	1.56
	Separated	5	3.91
	Never been married	11	8.59
	In unmarried couple	6	4.69
Financial Stress			
	Never	5	3.91
	Rarely	37	28.91
	Sometimes	51	39.84
	Often	28	21.88
	Always	7	5.47
Hispanic/Latinx Identity			
	No	123	96.09
	Yes	5	3.91
School/Degree			
	8^th^ grade or below	1	0.78
	Associate	6	4.69
	Bachelor's	40	31.25
	Master's	67	52.34
	Professional	1	0.78
	Doctoral	13	10.16
Shift			
	Day	110	85.94
	Night	3	2.34
	Rotating	3	2.34
	Don't work shifts	12	9.38

## 4. Discussion

Mindfulness in Motion has demonstrated significant benefit for HCWs in prior studies, and this study continues to support significant reductions in burnout and perceived stress, along with significant increases in resilience and work engagement for the nursing population [20]. We analyzed results from the subset of registered nurses and advanced practice nurses within HCWs compared to the larger group of HCWs. The results for both groups were nearly identical with all measures reporting statistically significant differences in pre to post intervention. The most notable difference between nursing subset and others in the HCW group is that the larger HCW group had a 26% reduction in burnout, while nursing staff had a 36% reduction in burnout. This 10% difference in the reduction of those who met burnout criteria post intervention suggests that nursing staff may experience greater benefits from the program as compared to other HCW professions. This may be due to the power of adopting a mindful perspective while performing their clinical work, possibly a different mindset than non-clinical MIM participants who do not work directly with patients and their families.

These results propose MIM as a unique strategy to support the individual mediating factors of the *Systems Model of Clinician Burnout and Professional Well-being*, while concurrently addressing the impact of the HCW feeling valued by the employer [11]. The system factor impacting burnout may be the financial commitment that the healthcare institution made in providing MIM free of charge to their HCWs, showing their value in the larger system. Regarding individual mediating factors, participants learned how to identify stressors, employ emotional regulation, cultivate effective sleep habits, and accept the uncontrollable. MIM taught nursing staff techniques to manage stress and build resilience, as a mediating individual factor encouraging professional well-being, rather than clinician burnout. The 36% reduction in burnout among the nursing group following completion of the MIM program may reflect the combination of obtaining these individual skill sets in addition to working for an employer that values their wellbeing.

These results show the effectiveness of mindfulness programming in nursing staff at a large academic institution in the Midwest. Many nursing challenges, such as short staffing, difficult patient interactions, and demanding hours are universal, which demonstrates that the themes and techniques taught in the program could assist other nurses in achieving professional and personal well-being. Nursing staff felt valued by the institution providing the MIM intervention and other institutions could adopt MIM or other evidenced-based well-being programming as a demonstration of employee value. Currently delivered across the US and in other English speaking international locations, MIM may be a uniquely effective intervention for implementation among nursing staff across the US and globally as it integrates easily into the clinical workflow.

Researchers will need to examine the extent to which enhanced HCW well-being benefits patients and the larger healthcare organization. Financially supporting and encouraging nursing staff to engage in interventions such as MIM may reduce nursing turnover and the number of sick days utilized by staff. These outcomes can have a positive impact on the organization, which could improve retention, as nurses may desire to work for an organization that values their well-being and encourages them to prioritize their mental health.

### 4.1. Implications for nursing burnout

To expand MBIs such as the MIM program to reach greater number of nursing professionals, researchers must consider providing interventions that are accessible, acceptable, and can be integrated into busy clinical workflows. Qualitative feedback from nurses who registered to participate in the MIM program, but were unable to commit to completion, revealed that difficulty finding time within their work schedule for wellness activities was the greatest barrier to participation. Mindfulness in Motion participants have repeatedly indicated their preference for readily available practices that can be easily incorporated into their workday. We transitioned MIM participants from using a website to access individual practices to a mobile smartphone application, designed specifically to support on-demand content and experiential practices. Mobile mindfulness apps have proven to be effective and feasible in nursing populations, as they enable quick meditations or breathing practices to be completed during the clinical day [34]. The smartphone format provides opportunities for accessible practices to be utilized during work and personal time. Mobile application use was encouraged throughout the program. The mobile app practices are two to five minutes each. Previously, the training website required a greater time commitment by requiring individuals to “log on” each time to access content. Additionally mobile app daily reminders via push notifications contribute to greater adherence. Researchers aiming to implement MBIs for nursing professionals working in the clinical environment could consider delivering their interventions via a mobile app, which may provide employees easy access to regularly practice a variety of mindfulness, breathing, and physical exercises while leading to greater reductions in stress and sustainability of program benefits.

### 4.2. Implementation to the clinical workflow

One of the most crucial components to advancing nurse well-being is integration of the programming into their clinical workflow. The Social Ecological Model (SEM) describes the components that can influence one's behavior [35]. There are five levels of the model: Individual, interpersonal, organizational, community, and policy/laws [35]. Within a large healthcare organization, system changes occur at the organizational level, which is defined as how the workplace, medical, or educational institution, and other organizations we are a part of influence our behaviors [35]. The highly significant changes in burnout, perceived stress, resilience, and work engagement speak to the benefits of MIM on the individual level of the SEM, but the qualitative feedback reflects a need for action at the organizational level. The qualitative feedback from nursing staff has emphasized the need for leadership support to promote well-being for all staff. If MIM programming can be supported at this level, mindfulness techniques and practices could be implemented seamlessly into the clinical workflow.

**Table 2.** Quotes on the Value of MIM at the Organizational Level.

Quotes on the Value of MIM at the Organizational Level.
*Incorporating practices/education into nursing units would be very helpful for nursing staff. They are very stressed and busy. If we could give them these tools I believe there would be a direct impact on patient care, satisfaction of nurses and patients and quality! Happy relaxed nurses=happy safe patients!??*
*Incorporating MIM to all new staff orientation and having all existing staff take MIM would support a healthy workplace, decrease stress-burnout-turnover, and improve job satisfaction, one's health, increase focus and increase job performance*.
*I think our staff is craving a more mindful work environment, we just need more staff and resources to accomplish this task. If the goal is to retain staff and try to keep them for their careers, then we would save a lot of money in the long run on orienting all the new staff we have coming in regularly and our infection rates & readmission rates would like reflect how well we retain staff*.
*I think that the organization can help by making it easier for staff to attend classes like this. We need to support them not just by attending ourselves but by making it something they are aware of, and by protecting their time to attend*.
*As an organization, I believe that this would help reduce the risk of making errors or compromising patient safety*.

Additional factors influencing MBI implementation into the clinical workflow are administrative buy-in and leadership support. Organizations without implementation success have described a lack of leadership support, emphasizing the “culture shift” necessary to move organizational priorities forward was absent. With the vast evidence of clinician stress and burnout, convincing leaders in administrative positions regarding the need for wellbeing resources for nursing professionals may now be somewhat easier as COVID exposed the extent of clinician burnout and the implications for society if we do not attend to these important members of society. Engagement from leaders in the form of implementation of targeted evidence-based interventions and providing opportunities for staff to engage in well-being activities during the workday appears paramount to reducing nursing personnel burnout.

### 4.3. Limitations

One of the major limitations of this study was the lack of a control group. Another significant limitation that limits the generalizability of the study was that study participants were primarily white, educated females, likely familiar with the evidence on nursing stress and burnout. As the researchers work to expand MIM to other populations and environments, consideration to racial, ethnic, cultural, and educational factors will be necessary.

## 5. Conclusion

Prioritizing the health and well-being of nurses and developing strategies to improve individual and organizational outcomes is essential to cultivating healthy and safe work environments. Mindfulness in Motion is an intervention that has been shown to be effective, delivering significant benefits for nurses and other HCWs. Interventions such as MIM that examine the psychosocial work environment can be instrumental in advancing the well-being of nursing professionals. Making available the MIM program through organizational sponsorship demonstrates an organizational commitment to employee health and well-being and has been shown to be especially effective in reducing burnout for nursing personnel.

## Use of AI tools declaration

The author declares no Artificial Intelligence (AI) tools have been used in the creation of this article.

## References

[1] World Health Organization (2019). Burn-out an “occupational phenomenon”: International Classification of Diseases.

[2] Shah MK, Gandrakota N, Cimiotti JP (2021). Prevalence of and factors associated with nurse burnout in the US. JAMA Netw Open.

[3] Dyrbye LN, West CP, Leep Hunderfund A (2020). Relationship between burnout and professional behaviors and beliefs among US nurses. J Occup Environ Med.

[4] Dyrbye LN, West CP, Kelsey EA (2021). A national study of personal accomplishment, burnout, and satisfaction with work–life integration among advance practice nurses relative to other workers. J Am Assoc Nurse Pract.

[5] Khamisa N, Oldenburg B, Peltzer K (2015). Work related stress, burnout, job satisfaction and general health of nurses. Int J Environ Res Public Health.

[6] Mäkinen M, Jaakonsalo E, Saarivainio R (2024). The effects of mindfulness training for emergency department and intermediate care unit nurses. Appl Nurs Res.

[7] Abdi F, Jahangiri M, Kamalinia M (2023). Developing a model for predicting safety performance of nurses based on psychosocial safety climate and role of job demands and resources, job satisfaction, and emotional exhaustion as mediators. BMC Psychol.

[8] Paskarini I, Dwiyanti E, Syaiful DA (2023). Burnout among nurses: Examining psychosocial work environment causes. J Public Health Res.

[9] Van Wert MJ, Gandhi S, Gupta I (2022). Healthcare worker mental health after the initial peak of the COVID-19 pandemic: A US medical center cross-sectional survey. J Gen Intern Med.

[10] Brouwer KR, Melander S, Walmsley LA (2024). A mindfulness-based intervention for acute care nursing staff: A pilot study. J Holist Nurs.

[11] National Academies of Science, National Academy of Medicine, Committee on Systems Approaches to Improve Patient Care by Supporting Clinician Well-Being (2019). Taking action against clinician burnout: a systems approach to professional well-being.

[12] Shanafelt TD (2021). Physician well-being 2.0: Where are we and where are we going?. Mayo Clin Proc.

[13] Stillman M, Sullivan EE, Prasad K (2024). Understanding what leaders can do to facilitate healthcare workers' feeling valued: Improving our knowledge of the strongest burnout mitigator. BMJ Lead.

[14] Kabat-Zinn J (1990). Full catastrophe living: The program of the stress reduction clinic at the University of Massachusetts Medical Center. 1990.

[15] Dobkin PL (2008). Mindfulness-based stress reduction: What processes are at work?. Complement Ther Clin Pract.

[16] Epstein RM (1999). Mindful practice. JAMA.

[17] Kabat-Zinn J (1982). An outpatient program in behavioral medicine for chronic pain patients based on the practice of mindfulness meditation: Theoretical considerations and preliminary results. Gen Hosp Psychiatry.

[18] Kabat-Zinn J (2003). Mindfulness-based interventions in context: Past, present, and future. Clin Psychol-Sci Pr.

[19] Kabat-Zinn J (2015). Mindfulness. Mindfulness.

[20] Klatt MD, Bawa R, Gabram O (2020). Embracing change: A mindful medical center meets COVID-19. Glob Adv Health Med.

[21] Klatt M, Steinberg B, Duchemin AM (2015). Mindfulness in Motion (MIM): an onsite mindfulness based intervention (MBI) for chronically high stress work environments to increase resiliency and work engagement. J Vis Exp.

[22] Klatt MD, Buckworth J, Malarkey WB (2009). Effects of low-dose mindfulness-based stress reduction (MBSR-ld) on working adults. Health Educ Behav.

[23] Steinberg BA, Klatt M, Duchemin AM (2017). Feasibility of a mindfulness-based intervention for surgical intensive care unit personnel. Am J Crit Care.

[24] Moffatt-Bruce SD, Nguyen MC, Steinberg B (2019). Interventions to reduce burnout and improve resilience: Impact on a health system's outcomes. Clin Obstet Gynecol.

[25] Klatt M, Marchenko N, Menser T (2018). Mindfulness in motion (MIM): a hospital intervention that reduces burnout and increases resilience for healthcare providers. Intern Med Rev.

[26] Duchemin AM, Steinberg BA, Marks DR (2015). A small randomized pilot study of a workplace mindfulness-based intervention for surgical intensive care unit personnel: Effects on salivary alpha-amylase levels. J Occup Environ Med.

[27] Malarkey WB, Jarjoura D, Klatt M (2013). Workplace based mindfulness practice and inflammation: A randomized trial. Brain Behav Immun.

[28] Klatt M, Westrick A, Bawa R (2021). Sustained resiliency building and burnout reduction for healthcare professionals via organizational sponsored mindfulness programming. Explore (NY).

[29] Maslach C, Jackson SE, Leiter MP (1997). Maslach burnout inventory. Scarecrow Education.

[30] Cohen S, Kamarck T, Mermelstein R (1983). A global measure of perceived stress. J Health Soc Behav.

[31] Connor KM, Davidson JR (2003). Development of a new resilience scale: The Connor-Davidson Resilience Scale (CD-RISC). Depress Anxiety.

[32] Schaufeli WB, Bakker AB, Salanova M (2006). The measurement of work engagement with a short questionnaire: A cross-national study. Educ Psychol Meas.

[33] Maslach C, Schaufeli WB, Leiter MP (2001). Job burnout. Annu Rev Psychol.

[34] Ball E, Rivas C (2021). Health apps require co-development to be acceptable and effective. Front Psychol.

[35] Bronfenbrenner U (1979). The ecology of human development: Experiments by nature and design.

